# Vertigo and Dizziness in the Elderly

**DOI:** 10.3389/fneur.2015.00144

**Published:** 2015-06-26

**Authors:** Lara Fernández, Hayo A. Breinbauer, Paul Hinckley Delano

**Affiliations:** ^1^Otolaryngology Department, Clinical Hospital of the University of Chile, Santiago, Chile; ^2^Otolaryngology Department, San Juan de Dios Hospital, University of Chile, Santiago, Chile; ^3^Clínica Alemana de Santiago, Facultad de Medicina Clínica Alemana, Universidad del Desarrollo, Santiago, Chile; ^4^Physiology and Biophysics Program, Institute of Biomedical Sciences (ICBM), Medicine Faculty, University of Chile, Santiago, Chile

**Keywords:** dizziness, presbystasis, vertigo, falls, elderly, aging

## Abstract

The prevalence of vertigo and dizziness in people aged more than 60 years reaches 30%, and due to aging of world population, the number of patients is rapidly increasing. The presence of dizziness in the elderly is a strong predictor of falls, which is the leading cause of accidental death in people older than 65 years. Balance disorders in the elderly constitute a major public health problem, and require an adequate diagnosis and management by trained physicians. In the elderly, common causes of vertigo may manifest differently, as patients tend to report less rotatory vertigo and more non-specific dizziness and instability than younger patients, making diagnosis more complex. In this mini review, age-related degenerative processes that affect balance are presented. Diagnostic and therapeutic approaches oriented to the specific impaired system, including visual, proprioceptive, and vestibular pathways, are proposed. In addition, presbystasis – the loss of vestibular and balance functions associated with aging – benign paroxysmal positional vertigo, and stroke (in acute syndromes) should always be considered.

## Introduction

The terms dizziness and vertigo cover a variety of symptoms regarding disorders of spatial orientation and motion perception, such as the illusion of rotatory motion (classical rotatory vertigo) or the feeling of unsteadiness, which can affect objectively the ability to achieve a stable gaze, posture, and gait (1). Altogether they represent a common and serious issue in the elderly, where its prevalence reaches 30% beyond 60 years of age (1, 2), while rising to 50% beyond 85 years (1).

The sole presence of dizziness in the elderly is a strong predictor of falls (3). Moreover, the presence of abnormal balance tests increases the risk of hip and wrist fractures (4). Injuries related to falls lead to mobility restriction and loss of independence, and increase the fear of falling, which also predicts subsequent falls (2). In addition, falls are the leading cause of accidental death in persons older than 65 years (5), while dizziness is one of the strongest contributors to the disability burden after age 65 (6).

Although the majority of these patients present benign balance disorders, (7–9), in the elderly, common causes of vertigo may manifest differently, with a more confusing constellation of symptoms, as patients tend to report less rotatory vertigo and more non-specific dizziness and instability than younger patients presenting with the same condition (9). Underlying this phenomenon is the progressive multimodal impairment of balance, including the loss of vestibular and proprioceptive functions, and the impairment of central integration of these and other sensory inputs associated with aging, which may also be called as presbystasis, presbyequilibrium, or multisensory dizziness (4, 7, 10). In addition, the skeletal muscle strength and mass are reduced with aging, increasing the risk of fall-related injuries in elderly patients (11).

On the other hand, a small number of patients harbor a serious and potentially life-threatening cause, mainly associated with stroke, and this risk of more serious diagnoses increases with age (12, 13). Altogether, vertigo, dizziness, and balance disorders in the elderly constitute a major public health issue, which needs adequate management by trained physicians. This mini review presents recent advances in the diagnosis and management of dizziness in elderly patients.

## Pathophysiology of Balance in the Elderly

Age-related degeneration of different neural structures affects balance, including the vestibular receptors, central vestibular neurons, cerebellum, and visual and proprioceptive pathways. The number of hair cells in the vestibular organs and the number of fibers in the superior and inferior vestibular nerves decrease with age (14–16). From a functional perspective, age-related deficits appear to be larger on semicircular canals, followed by saccular function, while the utriculus remains less affected (17–19). A steady asymmetrical decrease in the ability of sensing angular rotation with age has been reported, as assessed by video head impulse testing (vHIT) of the vestibulo-ocular reflex (VOR) (4, 19, 20). This fact is associated with a loss of dynamic visual acuity due to the inability to compensate fast head rotations with corrective eye movements, thus assuring a steady image over the retina (21). However, while on the acute phase of vestibular loss, this may cause intense rotatory vertigo (due to a sudden vestibular asymmetry), on elderly patients the slow onset of these chronic impairments would not manifest with vertigo. Instead, they complain about movement intolerance, instability, and insecure gait, particularly when sudden turns are needed, as there is an incapability of processing these movements properly. This may also explain the observed lack of rotatory vertigo in elderly patients with benign paroxysmal positional vertigo (BPPV) (8).

Nevertheless, while “active” vestibular symptoms may be milder or shifted toward instability, functional balance performance and disequilibrium phenomena are actually more severe. The sole presence of VOR asymmetry (which may present in elderly patients without history of an acute vestibular syndrome, and rarely in the form of bilateral vestibulopathy) is a significant predictor of falling (4, 22). In addition, compensation phenomena after vestibular loss are weakened in elderly patients, for example, impairment after vestibular neuritis is harsher on the elderly (23). Behind this lies degeneration of multiple non-vestibular subsystems. For instance, the medial vestibular nucleus, important in vestibular compensation due to its commissural fibers, shows lower neuron density in healthy older adults (24). There is also a mean loss of cerebellum Purkinje cells of about 2.5% per decade (25). Vibration and touch thresholds, the ability to detect position and direction of joint movements, and muscle strength also deteriorate with age (2). Visual accommodation, depth perception, and the ability to suppress nystagmus by visual fixation is diminished due to aging of the oculomotor system with increased saccade latency, and reduced eye tracking velocity (2).

Similarly, elderly patients with chronic pathological asymmetric vestibular evoked myogenic potentials (VEMPs) or deviated subjective visual vertical (SVV) tests, do not report dizziness or vertigo as significant symptoms, which may relate to central compensation occurring from the beginning of this slow onset of vestibular function (18, 19, 26, 27). This scenario leads to no pathological symptoms at all. Therefore, it is still controversial whether presbystasis by itself should be always considered pathological or not.

In summary, in order to maintain balance, the brain uses all available sensorial cues from vestibular, visual, and proprioceptive inputs, which in turn are integrated by the central nervous system to execute adequate motor responses. In this manner, age-related balance deterioration does not appear to behave as a unique standardized phenomenon, but the opposite, it seems to be extremely variable from patient to patient (17, 18, 20, 27). Moreover, minor new or acute impairments can affect disproportionately their capacity to cope difficult equilibrium scenarios, as every sensory modality may already be partially deteriorated. Current knowledge is moving toward determining which abnormalities in balance testing relate to higher risk of falling, and toward a balance disorder “profile” of selective impairments, which, as we propose, may guide a target-specific treatment (28–30). While asymmetric, severe, and multimodal balance impairments due to aging are likely to cause symptomatology *per se*, the magnification and distortion of the symptom spectrum of specific pathologies by presbystasis is perhaps more common. All these factors should be taken into account in the diagnosis and management of elderly patients.

## Diagnosis of Dizziness in the Elderly

Reaching a complete, meaningful, and treatment-oriented diagnosis in elderly dizzy patients remains an important challenge for even the most experienced clinician. Obtaining a good clinical history can be a tough task. It has been reported that more than half of elderly patients with balance disorders are vague, inconsistent, or contradictory in describing their symptoms (31). Besides, there is not a single symptom that can predict with specificity the underlying causes of dizziness, and most of the times, elderly patients have more than one cause of dizziness (32, 33). Moreover, caloric test responses depend on several factors that could be affected by age, such as ear canal volume, temporal bone thickness, and blood supply to the temporal bone (34). Several studies have found that caloric responses tend to increase in middle age with a peak between 50 and 70 years, and then decline modestly thereafter (35, 36).

A systematic assessment of balance should be achieved in this type of patient, for which recent technological developments are of great assistance. The impairment of each of the three semicircular canals can be examined by means of vHIT (37) procuring a reliable, objective, and quantitative value for VOR. Ocular and cervical VEMPs give equally reliable information over utricular and saccular function independently (38). The non-vestibular proprioceptive and visual sensory components of balance and their central integration in overall equilibrium performance can be thoroughly assessed by dynamic computed posturography (39). Altogether these tests provide an objective assessment of every component and subsystem of balance, allowing specific profiling of patients (40, 41).

Besides HIT, the SVV bucket test and modified Romberg and Fukuda tests represent low complexity alternatives for the same assessment, and may be used to develop simple, low cost, and quick screening procedures (20, 42). SVV by means of bucket test may even provide sensible assessment of utricular components beyond VEMP contributions (27). Head-shaking nystagmus and dynamic visual acuity testing among others constitute bedside, fast, inexpensive, and easy to interpret vestibular tests for VOR (4, 7, 18). Testing for postural hypotension, joint position sense, and gait disorders can also contribute to assess non-vestibular components in a bedside low-cost manner, contributing to designing an integral but component-specific treatment.

A particular scenario exists in acute onset of severe dizziness or vertigo; an acute vestibular syndrome, where ruling out stroke is critical, particularly in the elderly. The HINTS assessment protocol (head impulse test, nystagmus directionality, and test of skew) can be performed at the bedside, with high sensitivity and specificity to diagnose stroke in an acute vestibular syndrome (43). This three-step bedside oculomotor examination has shown better sensitivity than early magnetic resonance imaging (MRI). MRI can give a false negative result in vertebrobasilar stroke (44), and is not always readily available (45). A full description of the management of acute vertigo in the elderly is beyond the scope of this mini review, further readings can be obtained elsewhere (13, 46).

Also, of note is positional testing for BPPV. This clinical entity accounts for one in every three causes of dizziness in the elderly. With a simple diagnosis–treatment scheme (even in the absence of rotatory symptoms), testing should be performed routinely (8). Consequently, to seek a precise diagnosis, it seems to be mandatory to obtain a good clinical history and perform thorough neuro-otologic bedside examination, including postural testing, while the majority of patients may benefit from vestibular tests, and stroke assessment protocols for an acute balance disorder.

## Etiology

The majority of diseases that cause dizziness in any age group become more prevalent in older individuals. This can be explained by the cumulative probability of exposure or by age-related changes that make the elderly more susceptible to these pathologies (47). A summary of the main causes of dizziness in the elderly is shown in Table 1.

**Table 1:** **Etiology of dizziness and vertigo in the elderly**.

Peripheral vestibular	Benign paroxysmal positional vertigo
	Vestibular neuritis
	Bilateral vestibular loss
	Late-onset Meniere’s disease or decompensation (2)
	Labyrinthitis
	Occlusion of the anterior vestibular artery (48)

Central nervous system	Vestibular migraine (49)
	Transient ischemic attack of vertebrobasilar artery (50)
	Stroke
	Neurodegenerative disorders (51)
	Downbeat and upbeat nystagmus syndromes (51)

Cardiovascular (2)	Arrhythmia
	Postural hypotension
	Congestive heart failure
	Heart valve failure

Medications (52)	Antihypertensive
	Benzodiazepines
	Hypnotics
	Anxiolytics
	Antiepileptic

Multimodal balance disorder	Presbystasis (10)

Others	Primary and secondary neoplasia (breast and prostate) (53, 54)
	Somatoform vertigo and psychiatric dizziness (55)
	Musculoskeletal system disorders
	Proprioception and somatosensory loss

## Management of Elderly Patients with Dizziness

**Figure 1:**
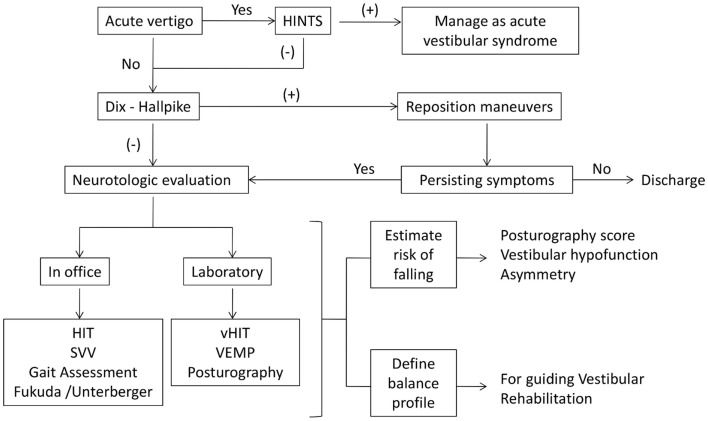
**Proposed flowchart for the management of dizziness in elderly patients**. An accurate anamnesis and physical examination will determine further vestibular, neurological, or cardiovascular tests. Patients with chronic vertigo should be evaluated with Dix–Hallpike maneuvers. After that, and depending on the available resources, office or laboratory tests help to estimate the risk of falling and define the balance profile to guide the management of these patients. On the other hand, every acute patient should be evaluated with the HINTS protocol.

As with younger patients, disease-specific therapies should be provided, such as repositioning maneuvers for BPPV and rehabilitation exercises for vestibular hypofunction. Nevertheless, special consideration is needed for elderly. A flowchart for the management of these patients is proposed in Figure 1. A high level of suspicion for BPPV should be maintained. In dubious cases, treatment attempts should be preferred, given diminished symptomatology and the safety and simplicity of reposition maneuvers (56).

In acute syndromes, stroke should always be ruled out by HINTS. Vestibular suppressants should be tapered quickly due to their inhibitory effect on central compensation (57). Although steroids have been proven to diminish functional loss over time, they may not contribute to acute symptomatic relief (58). Steroids side effects should be carefully considered before administration, particularly on this age group.

Current knowledge advises the initiation of vestibular rehabilitation (VR) as soon as possible after an acute vestibular syndrome (29, 30). VR works as a catalyzer and enhancer of central compensation on the basis of three principles: adaptation (rearrangement of VOR networking), substitution (strengthening of non-vestibular components of balance), and habituation (increase of sensory thresholds).

Chronic dizziness derived from previously acquired vestibular loss (vestibular neuritis, bilateral vestibulopathy among others) has good results with VR, particularly in terms of independence and quality of life, although it may need longer and more intensive therapy (28, 59). Moreover, VR is indicated in presbystasis, whereas the objective is to reduce symptoms or decrease the risk of falling (29, 30, 60). In addition, if there are deficits in lower extremity muscle strength, specific therapies directed to locomotor dysfunctions should be indicated (61). Proper balance characterizations may help in designing more specific and efficient interventions. For instance, a patient lacking postural stability will require postural- and gait-focused therapy. Care should be taken in focusing therapy on ongoing symptoms rather than solely on testing abnormalities, as certain patients could require other treatments prior to benefit from VR, such as in the case of vestibular migraine, or visually induced dizziness, among others.

Importantly, spontaneous compensation strategies differ among patients (half of the population tend to rely on visual cues, while the other half rely on postural information), supporting the need for customized rehabilitation programs (30). Computerized dynamic posturography seems to allow such characterization, while being a reliable objective measurement of the “amount” of unbalance and risk of falling, and monitoring progress (30).

Initiatives using Internet resources and mobile devices to support adherence and the realization of rehabilitation exercises at home have been developed (60, 62). Other balance-improving treatments being currently explored include biofeedback devices worn all day, which give tactile or acoustic cues when the center of gravity is being lost, allowing the patient to react accordingly (63). In severe cases of bilateral VOR loss and inadequate compensation strategies, the role of vestibular implants (devices similar in their concept to cochlear implants) is beginning to be explored, and interventions have already been made in the first patients with satisfying functional outcomes (64).

## Conclusion

Dizziness in the elderly remains a difficult subject, given the underlying factor of vestibular impairment due to aging in the form of presbystasis. The diagnostic and therapeutic approach must be multi-systemic and oriented to the visual, proprioceptive, and vestibular systems. BPPV and stroke (particularly in acute syndromes) should always be considered, given the frequency of the first and the severity of the latter.

Current vestibular testing allows a complete characterization of balance function and its deficits, and is becoming useful as a guide to planning treatment, where a cause-specific pathology is present, or presbystasis is the sole issue. Under this last condition, VR should be considered in the elderly where no other plausible balance disorder is suspected, in order to treat a probably symptomatic presbystasis. Here, resolution of symptomatology would confirm the assumed working hypothesis of presbystasis, while lack of progress would lead to further exploration of less common causes.

Future challenges on the subject include the further determination of vestibular impairment profiles and their specific VR alternatives, in order to achieve the shortest and most efficient therapy possible. However, research should also focus on preventive efforts to avoid falls. The threshold between what may be considered non-significant vestibular abnormalities and those correlating with a higher risk of falling should be better explored. This will inevitably lead to the establishment of a reasonable battery of (hopefully, bedside, low-cost, easy to interpret) examinations designed to rule out unacceptable risk for falling, in the fashion of the HINTS protocol for stroke.

## Conflict of Interest Statement

The authors declare that the research was conducted in the absence of any commercial or financial relationships that could be construed as a potential conflict of interest.
